# Perceived Barriers to Digitising School-Based Obesity Intervention: An Exploratory Study

**DOI:** 10.21315/mjms2022.29.4.10

**Published:** 2022-08-29

**Authors:** Norhasniza Yaacob, Ruzita Abd Talib, Amirah Ismail, Mohd Izwan Mahmud

**Affiliations:** 1 Center for Community Health Studies (ReaCH), Nutrition Programme, Faculty of Health Sciences, Universiti Kebangsaan Malaysia, Kuala Lumpur, Malaysia; 2 Center for Software Technology and Management (SOFTAM), Faculty of Information Science and Technology, Universiti Kebangsaan Malaysia, Selangor, Malaysia; 3 Center of Education and Diversity, Faculty of Education, Universiti Kebangsaan Malaysia, Selangor, Malaysia

**Keywords:** obesity module, case study, success factors, barriers factors, design technology

## Abstract

**Background:**

Schools serve as a focal point in which to engage students and school communities, including teachers, parents and other healthcare providers (e.g. nutritionists), in implementing obesity interventions. Bringing them together is beneficial to ensuring healthy eating among schoolchildren and to creating healthy school environments. However, there are barriers and success factors related to the implementation of such an intervention. Therefore, this study aimed to explore the barriers and success factors to school-based obesity interventions to improve obesity implementation.

**Methods:**

An exploratory case study with multiple units of analysis was conducted, including interviews, document analyses and a survey. The interview sessions involved six participants, including teachers and nutritionists. The survey was conducted among 121 participants involving 30 teachers, 78 parents and 13 school canteen operators. Pattern-matching techniques were employed to formulate the results.

**Results:**

The barriers consisted of the school system, parental awareness, staff availability, and children’s compliance. The success factors included the teachers’ role, the nutritional approach used, compliance with the healthy school canteen guidelines, and strong collaboration with the Parent-Teacher Association (PTA) and other agencies.

**Conclusion:**

It is necessary to digitise an obesity intervention module with some improvements, including strengthening the broader school community’s role and integrating nutrition education with technology to improve implementation.

## Introduction

The overall rate of obesity among children worldwide tripled from 1975 to 2016 (1). Although the rate of obesity in adults is higher than that in children, childhood obesity is increasing at a faster rate than in adults (2). In fact, almost one out of every five children and adolescents aged 5 years old–19 years old is overweight or obese (3). Furthermore, in 2017, about 38.3 million children under 5 years old were diagnosed as obese and overweight (4–5).

Childhood obesity increased by 48% in Southeast Asian countries between 2010 and 2016 (5). Malaysia, in particular, experienced a growth rate of 9.2% in childhood obesity from 2006 to 2019 (6). While the prevalence of childhood obesity did not change much in most developing countries, it increased at a high rate in developing Asian countries (7–8). As most Asian countries are transitioning toward a developed nation status, this has led to changes in lifestyle patterns and obesogenic environments affecting all ages (2, 9–10).

A healthy environment is critical in controlling and developing healthy habits among overweight and obese children. Over the years, the World Health Organization (WHO) has strengthened policies to regulate the promotion of foods and beverages, including restricting the advertising of foods high in salt and, saturated and trans fats as well as high-sugar meals and beverages in all settings, including schools (11).

Children learn at school the knowledge and experiences that shape their adult lifestyles. Thus, schools must regularly promote healthy lifestyles, including nutrition information and physical activities, among students (12). Schools can also actively reach out to family members and their communities as they support healthy behaviours among children, thereby improving the lives of other family members and the rest of the communities (13).

A systematic review of 60 studies on interventions used to manage childhood obesity found that in the preceding 5 years, the majority of countries, including Malaysia, emphasised school-based interventions, followed by community- and family-based interventions, and therapeutic interventions (14). However, interventions focused solely on nutrition education decreased, as this component was integrated into multi-setting and multi-approach school-, family- and community-based interventions. Hence, schools play a critical role in preventing childhood obesity.

While implementing obesity interventions in schools, stakeholders must ensure that these approaches are effective in reducing obesity. For this reason, the health promoting school (HPS) framework has been introduced. Schools under the HPS framework are defined as those that consistently build up their functions as healthy settings for social activities and learning (15). The dynamic and flexible implementation is carried out by improving school and national capacities; adopting an evidence-based and standards-driven approaches; increasing collaboration between the health and education sectors; and engaging a broader set of stakeholders, including parents, local governments and civil society organisations (16).

The HPS framework applies to whole-school approaches on: i) a health curriculum or discrete health interventions that encompass the entire school curriculum; ii) the broader ethos and environments of the schools; and iii) the engagement of parents, families, and the wider local communities (13, 17–18). It can be applied at any stage and one or more of the standards can be implemented.

The whole-school approach includes social support, multidisciplinary health and nutrition education and changes in healthy school environments aimed at improving the effectiveness of an intervention. Multidisciplinary approaches by school communities involving teachers, parents, school canteen operators and other healthcare personnel (e.g. nutritionists) play a critical role in obesity intervention, as they can improve the school environment and change children’s eating habits (19–21).

How does this whole-school approach work? In answering this question, the success factors and barriers in past obesity interventions must be identified to reduce the gap and improve interventions over time. It is also important to ensure their effectiveness and long-term sustainability (22). Such barriers and success factors are often related to the children themselves, their parents and families, implementing groups (e.g. health agencies), culture, school environment factors (e.g. canteens and physical activity implementation facilities) and various social aspects (23).

Furthermore, there is a need to explore the opinions of teachers who are involved in implementing obesity interventions. As teachers typically support intervention programmes, listening to their opinions and suggestions is critical to their effectiveness and long-term sustainability (24). Teachers also play a critical role in educating children about proper eating habits. Thus, multi-component interventions delivered by schoolteachers are likely to be effective (25). However, one limitation of teacher-delivered interventions is the lack of time or teachers’ inability to participate extensively in the interventions (13).

Parents also play a crucial role in intervention programmes to curb childhood obesity. Their perceptions of and knowledge about barriers to a healthy lifestyle, as well as the challenging factors preventing their participation in school-based interventions, are important considerations (22).

Schools must likewise play an active role in shaping environmental changes that, in turn, impact behavioural changes among children. A previous study suggested that improvements in school food environments may enable students to make healthier food choices and lower their body mass index (BMI) levels (26). An example of such an improvement is ensuring that healthy food and beverages are served to schoolchildren. Hence, all stakeholders within school communities, namely, teachers, parents and school canteen operators, play significant roles in inducing environmental changes and educating children about healthy eating.

Past experiences in implementing obesity interventions involving school communities should be explored further to obtain meaningful insights for improving future programmes. In particular, the problems, success factors and obstacles related to these interventions should be identified in the target group before improving implementation (27). However, the main problem to be resolved in the intervention approach involving the school community is how the delivery and guidance of nutrition-related knowledge is implemented because there are barriers to the participation of some stakeholders in school-based obesity interventions (28). If these barriers are overcome, such knowledge can be translated into changes in practices and attitudes toward healthy eating, which in turn, become a critical component of the effectiveness of nutrition education for children, particularly those who are overweight or obese (29).

In 2020, many countries’ healthcare and education systems were impacted by the COVID-19 pandemic. As a result, movements were restricted throughout the world to curb the spread of the COVID-19 virus and its variants. As physical and social activities were limited, the lifestyles of individuals also changed. Due to these restrictions, children’s health was impacted, leading to an increase in obesity (30). Furthermore, COVID-19-related school closures have had significant social and economic impacts, including disruptions in learning, poor nutrition, childcare gaps, unanticipated strain on healthcare systems, increase in school dropout rates and social isolation (31). Indeed, the education of children, including nutrition education, has undergone an extensive transformation as schooling shifted online (32–34). Teachers are required to find online content for nutrition education and even develop the content themselves if they cannot find adequate materials online. In addition to students, parents also need online guides so that they can practice good nutrition habits at home. School canteens also need online nutrition guides to plan healthy menus when they re-open after the pandemic. Thus, schools must improve their capabilities in digital technology to respond well to online learning requirements.

Knowledge from previous interventions is also critical in improving the effectiveness of ongoing and future obesity interventions. Thus far, available reports have focused on quantitative analysis by examining anthropometric measurements, improvements in children’s eating habits, health-related quality of life, and school performance. However, there is a lack of evidence from an in-depth exploration of obesity through a multi-setting implementation. Thus, a rigorous study of success factors and barriers is required to improve obesity management in schools. Such a study should focus more on educational interventions enhanced by technology in providing guidance and obesity intervention modules, which can be used by school communities to overcome existing intervention barriers.

### Scope and Key Contributions of the Study

This study aimed to explore the barriers and success factors, as well as provide insights from the perspectives of nutritionists and school communities, particularly school administrators, teachers, parents and school canteen operators, who are involved in school-based obesity intervention programmes. We also highlight the need to digitise an obesity intervention module to improve and sustain such school-based programmes.

## Methods

### Study Design

This study employed qualitative research that uses an exploratory single-case study, in which multiple units of analysis examine a single, current-day occurrence or action within a constrained setting. Multiple empirical data were obtained through various approaches, as guided by Yin (35). A thorough investigation is required using empirical data obtained over time from a well-defined case to explain the phenomenon’s context and processes (36). The objective of a case study is to conduct in-depth research on a specific subject, such as an individual, a group, an institute or a community, to help identify essential aspects, processes and relationships involving the study subject.

To meet the current study’s objectives, the selected case study was based on current obesity intervention programmes for obese primary school-aged children. An exploratory single-case study with pattern-matching technique analysis was used in this article because the variables contributing to the research problem and objective of the study were largely dependent on the interventions themselves. However, the main problem in school-based obesity interventions was contextual, complicated and multifaceted, often involving multi-setting and multi-component approaches. The complexity of this situation resulted from the size of obesity interventions conducted in Malaysia. However, as the obesity rates among children continue to rise, the ensuing significant disparities must be investigated.

The nutritionists who serve as programme coordinators and implementers, along with school administrators and teachers, were included in the data-gathering stage. The parents of obese children and school canteen operators were also involved, as the researchers could use qualitative case study methods to investigate complex phenomena in a specific context (36). This approach also enables a more in-depth assessment of the success factors and challenges in implementing a programme (37).

### Case Study Context

An exploratory single-case study context on the obesity intervention programmes is presented in Table 1 below.

### Study Participants and Sample

The sample was obtained using the purposive sampling method. A total of 12 schools that participated in obesity intervention programmes from 2017 to 2019 were chosen as part of the case study. These schools were under the jurisdiction of the Education Department of Kuala Lumpur. Screening was carried out to identify participants who met the study’s inclusion criteria.

A total of six participants, three nutritionists and three school administrators (head schoolteachers, senior assistant student affairs and health committee teachers) were selected to participate in the in-depth interview based on their experiences in programme implementation. The snowball sampling technique was used to recruit the participants for this interview purposes.

The convenience sampling method was then used with survey participants in the next stage of the analysis. Overall, a total of 121 participants - 33 teachers, 78 parents and 13 school canteen operators - were involved. A set of self-administered questionnaires was used to determine the perceptions of the survey participants regarding school-based obesity interventions.

### Data Collection and Instruments

This study employed three stages of adaptive analysis: i) in-depth interviews; ii) document analyses and iii) a survey guided by hierarchical modeling by Trochim and Donnelly (38) and the multiple units of analysis by Yin (35).

#### Stage 1: Interviews

Semi-structured questionnaires were used during our interviews with the school administrators, teachers and nutritionists. Each interview lasted for approximately 30 min–60 min. The participants were encouraged to be comfortable and speak freely regarding their experiences with the interventions. Three key research questions were used involving the following: i) factors that contributed to the success of the school-based obesity intervention; ii) the barriers that hindered the implementation of the interventions and iii) suggestions to improve the interventions and the need to digitise the obesity intervention module for the school community (consisting primarily of teachers, parents and school canteen operators) to be used for future school-based obesity intervention programmes. All three research questions were based on the HPS framework used to conduct interventions in schools and references from past studies (28, 39–41).

The semi-structured questions also underwent an expert validation procedure, including discussions with supervisors and narrative reporting of modifications and improvements. However, the researchers remained open and flexible throughout the research to obtain rich and nuanced findings that encompass and explain the complexity of real-world situations. For example, new thoughts or insights from participants may suggest potentially productive lines of inquiry or a detailed analysis of an account may uncover minor discrepancies that require further exploration.

All interview sessions were recorded using a Sony voice recorder and a Canon Power Shot SX 430IS video camera. Field notes were also taken to record essential information during the session.

#### Stage 2: Analysis of documents

All documentations relevant to the implementation of the obesity intervention were gathered, which consisted of textual and visual activity reports for completed activities/modules, activity outcomes, and records of food and beverage sales from the school canteen before, during and after the programme. The data were then analysed using descriptive and content analyses.

#### Stage 3: Survey

A survey of parents, teachers and school canteen operators was undertaken to seek their feedback and perceptions on the effectiveness and challenges of implementing and supporting school-based obesity programmes for children, as well as the need for the development of an obesity module as a key reference for use in future programmes. Self-administered questionnaires were used in conducting the survey and SPSS Statistics version 22 was used to analyse the data obtained from the questionnaire in descriptive statistics.

The findings from the document analysis and survey were merged with the interview data for triangulation purposes. The NVivo 12 application was utilised to organise the three types of data collected, resulting in the identification of well-formed themes as the main outcomes.

## Data Analysis

### Analysis Procedures

Content and thematic analyses were applied at the first stage of the data analysis, guided by Braun et al. (42), followed by flexible pattern matching to summarise the findings. It consists of seven steps: i) data transcribing; ii) data familiarisation; iii) initial code generation; iv) theme review; v) theme definition; vi) theme naming and vii) reporting of findings.

### Pattern-Matching Principle

Flexible pattern matching uses a conceptual framework from content and thematic analyses to provide an overview of the results obtained after investigating the success factors, barriers and suggestions for improving the interventions. Further, the findings on the need to digitise an intervention obesity module for the school community were strengthened. These were aligned with the goal of flexible pattern matching to determine how inconsistencies and breakdowns emerging from the process can be problematised, how theoretical ideas can be developed, how consistency can be applied and how the contextual boundaries of existing theories can be expanded (42–43).

Flexible pattern matching involves the iterative matching of theoretical patterns derived from the literature and observed patterns emerging from empirical data (44). In the current study, empirical/observation data refer to interviews, document analyses and surveys that have been conducted under chosen case study. Meanwhile, the theoretical patterns were derived from the existing HPS framework. The research team studied the existing patterns that emerged from enforcing the current HPS framework and compared them with the observed patterns collected through empirical data. The two sets of emergent patterns were then matched to map the similarities and differences between the actual practice of the interventions, with the exploration of barriers that may lead to meaningful findings. In turn, this can help bridge the gap and improve school-based obesity interventions. Furthermore, this approach is ideal for exploration and theory development. Thus, rigor is combined with a high level of flexibility to identify and describe patterns as accurately as possible (45). Hence, in this study, the pattern-matching process application was used to summarise the case study findings in the form of a conceptual framework.

### Validity and Reliability

In a qualitative inquiry, such as a case study, the quality of the findings becomes an issue. The researcher’s interests and beliefs may impact the interview process. Thus, in the current study, we employed a four-step procedure to ensure that we obtained high-quality data: triangulation, member check, audit trail and peer examination (46–50).

## Results

The characteristics of the study participants and the documents analysed are presented below.

### Characteristics of Participants for Interview Analysis

A total of six interview participants had a minimum of 7 years and a maximum of 30 years of experience in their respective fields. The participating teachers accumulated between 10 years and 30 years of experience coordinating the nutrition intervention programmes in their respective schools. The nutritionists had 6 consecutive years of experience conducting nutrition intervention programmes from 2016 until the present. Based on this profile, the participants were able to draw on their extensive experiences to provide information as to what was required to improve future school-based interventions.

### Document Analysis Characteristics and Summary Reports

All supporting documents were divided according to three criteria, as presented in Table 2.

The first criterion involved documents measuring the programme’s effectiveness consisting of students’ anthropometric data and their levels of knowledge before and after the intervention. The significant findings were revealed as supporting material for the success factors of the interventions.

The second criterion consisted of activity reports throughout the interventions. The findings showed that more improvements were needed regarding time constraints and parents’ involvement. The teachers also needed to reshuffle the interventions, as they needed to focus more on their schools’ respective curricula rather than on health and other co-curricular activities.

The final criterion involved the assessment of environmental factors. We also included data from the compliance with healthy food and beverage sales in school canteens as part of the analysis. Many previous studies have suggested the importance of including environmental factors in an obesity intervention. Compliance with healthy school canteen guidelines throughout the programme implementation has been revealed as one of the success factors. However, the low compliance rate recorded after 1 year demonstrated the challenges involved in maintaining the programmes’ efficacy. Hence, the document analysis revealed many success factors and barriers during and after the programmes.

### Survey Reports

The survey focused on the participants’ perceptions of the programmes’ effectiveness, as well as the need for improvements in the development of digital intervention modules for teachers, parents and canteen operators to ensure that the obesity interventions can be implemented more effectively in the future. The demographic profiles of the teachers, canteen operators and parents are presented in Table 3.

A total of 121 participants involved 30 teachers, 13 school canteen operators and 78 parents agreed to answer the questionnaire provided. The teachers involved in coordinating the programme activities were selected by the school principal regardless of their position. This revealed that nutrition and health education, including obesity interventions, was a top priority for every school, with teachers actively promoting healthy eating habits among schoolchildren. The mean age and standard deviation (SD) of teachers were 36.4 and 6.1 years old, respectively and majority had a degree holder in education.

The mean age of the canteen operators was 43.2 years old and SD was 14.3. The majority graduated from secondary school (*n* = 12, 92.3%) and only one (7.7%) obtained higher education. The canteens were mostly managed by experienced operators/managers [8.9 (SD = 6.0)] with 5 years–10 years of experience (61.5%), followed by those with 10 years (23.1%) and less than 5 years (15.4%) of experience. Therefore, the canteen operators were experienced in both operating canteens and food preparation. Furthermore, all of them completed healthy cooking training and were familiar with implementing healthy school canteen guidelines.

A total of 78 parents answered the questionnaire. The mean and SD values for the participants were 42.4 (SD = 5.8) and 39.9 (SD = 3.9), respectively. From the parent’s characteristics, majority of them inclusively both fathers and mothers were working in public and private sectors as well as self-employed.

As mentioned previously, the questionnaires were used to summarise the agreed-upon points and factors to consider among the school communities regarding their perceptions of obesity intervention and the need to develop a digital obesity intervention module. This is consistent with the three principles of data collection for case studies: i) use multiple sources of data; ii) create a case study database and iii) maintain a chain of evidence (35). Thus, a summary of the survey findings is provided in Table 4.

The overall mean score of feedback regarding intervention effectiveness was higher for teachers and moderately high for both canteen operators and parents. These findings contradicted the interview results regarding the success factors of the intervention. It was due to, the nature of the survey study report based on quantitative data that more on the perception of teachers using the structured survey and the exploration was not conducted rigorously as interview sessions.

The parents’ and school canteen operators’ sites, on the other hand, revealed lower mean scores in some aspects. The lower values reported for the parents’ survey were 2.95 (SD = 1.04) and 2.96 (SD = 1.02), which resulted from the consumption of processed and high-calorie foods among their children due to parents were unable to prepare healthy food for their children due to lack of skills and time to prepared. In addition, other factors that contributed to lower means score for the parent’s perception regarding the intervention effectiveness was the presence of parents in any school activities. It was difficult for parents to join the programmes due to work commitments and daily duties. This supported one of the barriers identified during the subsequent exploration of the interview, thus confirming the need for digitising an obesity intervention module.

For school canteen operators, it was difficult to classify food categories as ‘to sell’ or ‘not to sell.’ A lack of menu modification ideas resulted in low mean scores and SD values of 2.07 (SD = 1.03) and 2.69 (SD = 0.95). Thus, school canteen operators need some guidelines to improve their knowledge and skill towards preparing a healthy menu to be sold.

Hence, it was demonstrated that intervention barriers must be overcome by including the people closest to the children, such as parents, teachers and canteen operators, in an effort to modify their eating habits. One suggestion was to have a digital obesity module to empower them in skills and knowledge regarding healthy eating in order to help them in facilitating and improving such good eating habits among children to help in improving interventions effectiveness. The overall survey findings were used as supporting data for the next theme formation phase of the empirical data.

### Themes identified from empirical-based data and their comparison with theoretical patterns

The themes from multiple sources of data comprising interviews, document analyses and survey are presented in Table 5.

## Discussion

### Success Factors

Four themes were identified from the exploration of success factors, as shown in Table 5. Among these, the nutritional component has been identified as a key factor in implementing effective obesity interventions for children (51). The findings of the current study showed that the implementation of nutrition activities was considered effective, as there were clear objectives, hands-on activities, and other activities to engage students and improve their confidence in adopting healthy eating habits. Children involved in the interventions could recall what they had learned. These findings were also supported by document analysis, for which the assessment of knowledge level was reported as significant after the intervention. However, it was challenging to turn acquired knowledge into good eating practices; in fact, the children’s acquired knowledge did not lead to a significant change in BMI. Thus, multi-component interventions, including exercise and specialised nutritional counseling for obese children and adolescents, are critical for significant changes in adiponectin levels, BMI and waist circumference in controlling childhood obesity (52).

Furthermore, teachers played an imperative role in ensuring that the interventions were delivered successfully. The teachers and other school administrators expressed full support for intervention management, including the provision of all the required components before and after the activities. Ongoing support can positively influence children’s eating habits and the school environment through social interactions (53–54). Another success factor was the strong collaboration between the Parent-Teacher Association (PTA) members and other agencies. Continuous support from PTA members was an added value toward obesity interventions, as they provided opportunities to participate in planning and exploring recommendations for better impact.

Finally, another success factor that emerged was compliance with the healthy food and beverages selling guidelines, which contributed to environmental changes. All the school canteens obtained the required score during the intervention period due to the comprehensive guidance given to them. Unfortunately, only 4 out of 12 schools maintained their compliance rate after a year of intervention. One canteen operator mentioned that the guidelines were useful in producing healthy meals for schoolchildren, but the latter did not prefer the specified food and beverages, as they were lower in sugar, fats, and sodium. Some of the canteen operators mentioned that they did not have the ability to prepare recommended nutritious food. These findings matched those of Chan et al. (55), whose work investigated the awareness, barriers, and facilitators of policy implementation in obesity management. The healthy food and beverages selling guidelines in school canteen are recommended by the government to motivate schools to promote healthy eating among children. However, most school canteens continue to fall short of the standards (20, 56). Therefore, the commitment of school canteen operators, ongoing guidance from teachers and nutritionists, and continuous monitoring are all mandatory components for maintaining the sales of healthy menu items and providing better food choices for students. In turn, this will help increase the effectiveness of interventions in schools. It must be noted that the healthy environment policy not only focuses on the sale of food in the canteens, but also on all other environmental aspects, such as facilities for implementing physical activities in schools.

The success factors identified in this work aligned with the HPS framework. It is considered a novel approach in conducting school-based intervention, as it integrates curriculum aspects, policy, and partnership, which are key factors in the successful implementation of intervention programmes. However, the implementation of the whole-school approach highlighted by HPS framework entails great effort and participation from the communities surrounding schoolchildren. Therefore, the challenges that hindered the implementation of interventions must be explored and mitigated to sustain long-term impacts.

### Barriers

Four barriers related to the implementation of the intervention were identified: school system, parental awareness, staff availability, and children’s compliances and attitude. School rules were determined to be the main challenge to implementing an effective school-based obesity intervention. As the findings revealed, the schools’ existing curricula and activities were already packed, and any additional intervention programmes burdened the teachers. Thus, the teachers needed to reschedule their timetables to accommodate the intervention, and those who were not originally involved in health education were also mobilised. The Ministry of Education Malaysia has prioritised formal teaching and learning in schools. Therefore, intervention activities are not encouraged during class time. However, it also depends on the schools themselves (57). Hence, the main challenges faced by teachers when implementing activities in schools must be understood to ensure the effectiveness of the interventions (41).

The second challenge was the lack of parental awareness, which included the parents’ lack of support and commitment and the constraints of providing healthy meals for their children. The results showed that some parents did not want their children to participate in activities other than formal learning, as they believed that doing so would interfere with their children’s academic achievement. Some parents, however, welcomed intervention activities but were unable to participate because of several constraints, such as the lack of skills and time to prepare meals and the inability to commit to any activities due to their busy work schedule. Parental involvement is crucial in any intervention, when parents were involved, children showed significant improvements in their eating behaviours. However, the further integration of different types of environments, such as more active parental involvement, might be factors that influence intervention effects on children’s eating behaviours (58). Thus, empowering parents to educate their children is critical in reducing barriers to the intervention’s effective implementation.

The third challenging factor identified was the availability of nutritionists who worked closely with teachers to carry out the intervention activities. Nevertheless, the results showed that the nutritionists and teachers worked together in everything they could to implement the programmes to the best of their abilities despite low staff availability and limited time. The practice in Malaysia is for teachers to teach children about nutrition and healthy lifestyle, regardless of their training and background. In Korea, the government hires school nutritionists to monitor and implement the provision of healthy school meals and to focus on guiding teachers and students. Hence, obesity interventions in Korean schools have been found to be more effective than in other countries (59–60). Therefore, multidisciplinary support is highly needed because obesity management focuses on providing education, managing the clinical aspects of obesity and bringing about healthy environmental changes (19). Thus, in Malaysia, more nutritionists, particularly at the school level, are urgently needed to provide regular support to teachers, parents and children and, close monitoring and improvements to a healthy school environment.

The final barriers identified were the children’s compliance with and attitudes toward healthy eating. Although the students were exposed to intervention activities, the influence of unhealthy foods was still an issue when implementing the intervention. This finding suggests that although the children learned about healthy food, their attitudes and desires to consume unhealthy foods remain unchanged. Therefore, more support from such communities as parents, teachers and peers, along with a combination of environmental factors, are promising strategies to increase the effectiveness of obesity intervention.

School authorities, teachers, parents, and students themselves who are active participants all play essential roles because the lack of interest on the part of school authorities has been identified as one of the significant factors in the absence of continuous nutritional and physical education in preventing childhood obesity (61). However, our study results showed strong support from school authorities for teachers and administrative staff, as well as enhanced collaboration with PTA members. Nevertheless, the interventions had to be altered and not arranged in a timely fashion owing to the time constraints of the teachers and the nutritionists. These issues directly affected the interventions. While past studies have shown less parental support and student participation, reaching out to parents and seeking their involvement significantly boosted the effectiveness of interventions (62). Many practical considerations have a significant impact on parents’ ability to participate in such programmes (63).

### Suggestions for Improvement

The consideration of success factors and barriers when implementing an intervention must be prioritised when making recommendations for improvement. Furthermore, intervention programmes in schools worldwide are frequently regarded as lacking coordination in their implementation due to programme barriers (64–65). Evidence has been provided to identify common barriers. In line with the HPS framework, a comprehensive approach when implementing intervention activities in schools requires re-orienting school systems toward health promotion and intervention. Such a re-orientation should include integrating health promotion, particularly information regarding healthy nutrition, into the school curriculum; making it a school policy to provide healthy physical and social environments; and involving parents and the wider community in school activities (66).

Overall, three themes emerged in this study, all of which were related to improvements arising from the HPS framework’s suggestions, namely, strengthening the roles of school communities (including parents, teachers, canteen operators and others), applying interventions in the school curriculum and using students as role models. Strengthening the role of school communities must adequately be addressed, as they play a significant role in improving the interventions (8, 67–69).

The next suggestion involved the integration of intervention components into the curriculum and co-curricular activities. A previous study employed the nutrition education curriculum used in the intervention to improve children’s knowledge and increase their preferences for fruits and vegetables (70). Although this is consistent with the current practice in Malaysia, there is growing interest in seeking more practices, further exploring the suitability of school and teacher times, and carefully revising the intervention components accordingly. The interventions used and the components integrated into a curriculum should not just improve knowledge but, more importantly, have a better impact on reducing the obesity rate. Thus, the next step involved using the student as an example to encourage peers or family members to develop a healthy lifestyle. Adolescents can influence family behavioural changes because of their ability to initiate discussions with parents regarding the nutrition they are learning and seeking to know (71).

A novel approach emerged from the findings of this study: integrating the use of technology in nutrition education. One of the requirements is empowering and guiding the school community as the primary party to trigger and be actively involved in the intervention. However, these barriers pose certain challenges. Thus, there is an urgent need to develop reference tools for obesity intervention modules, particularly in a digital format. A study has demonstrated that the effective implementation of HPS framework is a complex kind of intervention involving multi-factorial and innovative activities that are urgently needed in many domains, such as curricula, school environments and communities (34).

The use of technology in delivering nutritional information can overcome the barriers to implementing interventions. A systematic review found that technology-based interventions can enable overweight and obese children to manage their weight and improve their nutritional status (72). Other research focusing on technology and social media-based interventions for parents and children found that the effectiveness of the intervention is improved with the application of technology (73). At the same time, apart from strengthening teachers’ knowledge and skills, parents and canteen operators should also be given priority because these groups have a powerful influence on children’s nutrition at home and at school, respectively. Therefore, the management of school interventions involving teachers, parents and canteen operators must be supported as additional components, as the latter cannot just focus on the family and children only.

To date, there has not been a comprehensive technology-based intervention module emphasising all the essential elements of an obesity intervention that integrates school communities (teachers, parents and canteen operators). Technology can be used easily because it can be adapted to user-managed interventions to provide customised advice, which can help meet user needs and preferences (74). Moreover, the delivery of intervention elements using technology to facilitate interactions between programme coordinators and parents can help control children’s nutritional intake and monitor their weight gain status (75). Technology also serves as a medium through which teachers can have broad access to new teaching approaches and strategies while supporting the need for pragmatic programmes that fit the existing curriculum and school structure without posing additional burdens on teachers (76). Hence, technology-based methods are needed to provide nutrition guides for school communities (77).

During the COVID-19 pandemic, technology was used in many daily activities. During this period, many people used various digital technologies to convert and manage structural and cultural changes and barriers (78). Digital technologies have also been used extensively to transform the delivery of education from traditional to digitalised classrooms (32). Thus, by creating a technology-based obesity intervention module, teachers, parents and students will be provided with adequate support throughout the interventions. Triangulation results from the survey also support the need to establish a digital intervention module, particularly for school communities, to improve the intervention component.

### Conceptual Framework Derived from the Identified Matching Process

The findings were reported using a conceptual development framework. The presented data summarise the results of the studies.

**Figure 1:**
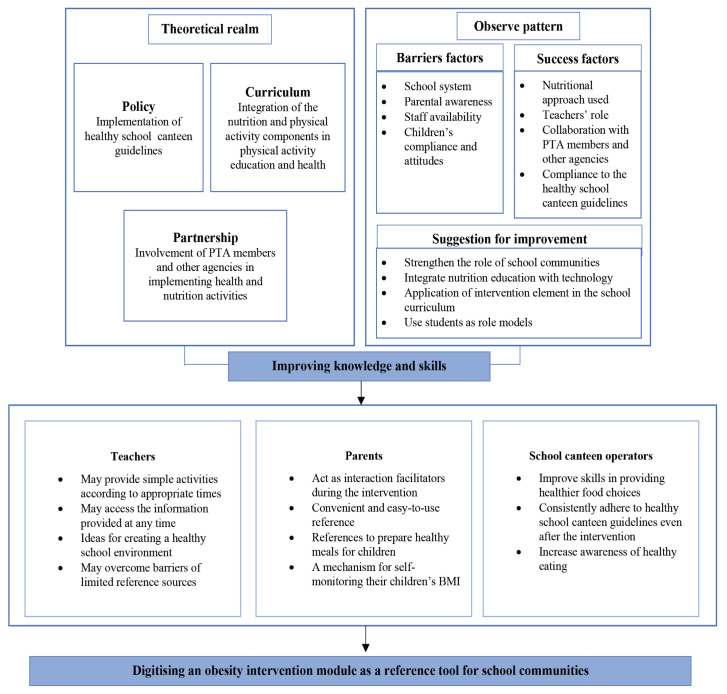
Conceptual framework: summary findings of pattern matching from the interview, document analysis and survey data

Exploring barriers and success factors has resulted in the creation of proposals for improvement, in line with an emerging new pattern based on the HPS framework, as presented in the conceptual framework illustrated above (Figure 1). This is presented in the theoretical realm and matched with the observed patterns discussed earlier. The articulation of findings from flexible pattern matching is structured by theoretical patterns or a theoretical framework that enables the comparison of theoretical and empirical patterns presented in detail (46). The exploration of barriers and success factors thus provides future directions for obesity intervention studies that aim to tackle the increasing rate of childhood obesity.

## Conclusion

A case study is a robust design that uses multiple methods and data sources to bring different perspectives together to provide a better understanding of the case under investigation. The difficulties in reconciling the multiple perspectives of knowledge in case study research can be overcome by using a pattern-matching technique. Thus, in this paper, we discussed a research-based example through a single-case study with multiple units of analysis, using a pattern-matching technique as a method of analysis. This work contributes to the literature by clarifying the steps in pattern matching using a practical application and by building methodological strategies for case study research. The development of a conceptual framework derived from this case study finding will bridge the gap in the implementation of obesity interventions and then examine the extent to which we have implemented the interventions effectively with a systematic approach.

## Limitations of the Study

One limitation of this study is that in-depth interviews were conducted only with teachers and nutritionists. This is because conducting a case study entails a significant amount of time, money, and researchers’ ability to work together as a team to complete the case study satisfactorily. Moreover, the sample may be limited, as the researchers had to conduct multiple interviews with the same participants and conduct validity checks to ensure that the collected data were reliable. Finally, assembling a team that can work well together posed difficulties as well.

## Future Research

Digital versions of the school community’s module guide can serve as reference tools to be used when implementing school-based interventions. Related to this, future studies should pay extra attention to the elements in the module’s intervention to meet the needs of teachers, parents, and canteen operators as key factors in effective intervention. In addition, future works must identify appropriate software and integrate technological theories in designing, developing and testing the usability of the digital obesity intervention module. This module should be able to improve users’ knowledge and abilities by providing convenient, accessible and user-friendly tools. Most importantly, the knowledge offered must be relevant and capable of influencing improvements in the management of childhood obesity.

## Figures and Tables

**Table 1:** Context of the case study (*n* = 12)

Intervention programme	Context
Duration of the programme	6-months
The total number of schools involved in the case study	12 schools (2017–2019)
Types of school involved	Primary school
Intervention module	Using the current module designed by the Nutrition Department Kuala Lumpur
Component of the module	Five nutrition topics, one session on physical activity and healthy school canteen assessment
Programme coordinator/implementer	Nutrition officers, school administrators and selected teachers and PTA members
Target changes	i) Obese and overweight children 10 years old–11 years old of age ii) Healthy school environments and canteens

Note: PTA = Parent-Teacher Association

**Table 2:** Document analysis reviewed (*n* = 12)

Type of document analysis explored involving schools (*n* = 12)			

		*n*	%
A) Reports on BMI and knowledge of obese and overweight children (*n* = 369) from 12 schools	A1: Boys	202	54.7
	A2: Girls	167	45.3

		**Mean**	**SD**

	A3: Age (years old) boys	10.62	0.49
	A4: Age (years old) girls	10.65	0.48

	**Significant result after intervention (** ***P-*** **value)**		

	A1: Overall BMI z-score	0.747	
	A2: BMI z-score for girls	*0.010	
	A3: BMI z-score for boys	0.800	
	A4: Knowledge on nutrition and physical activity	*0.003	
	
		***n***	**%**
	
B) Reports on activities (*n* = 12 schools)	B1: Involvement of parents during school-based activities	3	25.0
	B2: Teachers need to reschedule the activities	9	75.0
	B3: Time constraint in conducting activities	9	75.0
C) Records of compliance with healthy food and beverage sales in school canteens	C1: Compliance rate guideline during an intervention	12	100
	C2: Compliance rate after intervention	4	33.3
	C3: Regular monitoring after intervention by schools/nutrition officers	4	33.3

Notes:

* Significant based on *P* < 0.05 using paired *t*-test

**Table 3:** Characteristics of the survey participants (*n* = 121)

Teachers (*n* = 30) Age mean 34.6 (SD = 6.1)									

Gender	*n*	%	Position	*n*	%	Position		*n*	%
Male	13	43.3	Health teachers	6	20.0	Senior teachers		5	16.7
Female	17	56.7	Canteen teachers	7	23.3	Classroom teachers		6	20.0
			Club and sport teachers	6	20.0				

**Higher education background**	***n***	**%**	**Working experiences (as teachers)**	***n***	**%**	**Experience in conducting nutrition activities in school**		***n***	**%**

Diploma in Education	3	10.0	< 5 years	3	10.0	< 5 years		20	66.7
Degree in Education	24	80.0	5 years–10 years	1	3.3	5–10 years		10	33.3
Master in Education	3	10.0	> 10 years	26	86.7				

**School canteen operators (** ***n*** ** = 13)** **Age mean 43.2 (SD = 14.3)**									

**Gender**	***n***	**%**	**Education background**	***n***	**%**	**Experiences operating a canteen** **Mean (SD) = 8.9 (6.0)**			

								***n***	**%**

Male	3	23.1	Secondary schools	12	92.3	< 5 years		2	15.4
Female	10	76.9	Higher education	1	7.7	5 years–10 years		8	61.5
						> 10 years		3	23.1

**Parents (** ***n*** ** = 78)** **Fathers: age mean 42.4 (SD = 5.8)** **Mothers: age mean 39.9 (SD = 3.9)**									

	**Education background**					**Occupation**			

	**Fathers**		**Mothers**			**Fathers**		**Mothers**	

	***n***	**%**	***n***	**%**		***n***	**%**	***n***	**%**

Secondary schools	38	48.7	34	43.6	Public workers	38	48.7	32	41.0
Higher education	40	51.3	44	56.4	Private workers	25	32.1	21	26.9
					Self-employed	12	15.4	3	3.8
					Unemployed	3	3.8	22	28.2

**Table 4:** The perception among participants towards the effectiveness of the interventions and agreement on digitising the obesity module (*n* = 121)

Item/Questions	Teachers (*n* = 30) Mean (SD)	Canteen operators (*n* = 13) Mean (SD)	Parents (*n* = 78) Mean (SD)
i) Feedback on the effectiveness of the interventions from the participants’ perspective	4.42 (0.47)	3.14 (0.89) *2.07 (1.03) *2.69 (0.95)	3.34 (0.96) *2.95 (1.04) *2.96 (1.02)
ii) Agreement on the requirement of digital obesity module	4.42 (0.47)	4.36 (0.55)	4.23 (0.54)

Note:

* Lower mean was reported in two items

**Table 5:** Identified barriers and success factors, and suggestions for the improvement of obesity interventions

Empirical pattern			Theoretical pattern

Sub-theme	Theme	Verbatim	

Success factors			
Practical and hands-on activities Activities that interest the students Activities that meet the objectives Activities that increase students’ confidence	i) Nutritional approach used in the programme	***Teacher 2*** *My opinion for the healthy eating part, so far, the impact is what they can eat when we ask them in class, they can answer spontaneously, it means that children can know this and that and they can relate to things and situations. that we share. It means that after they followed the programme, they would know this is the right way to eat even though they are at the bottom of the class list, they got that knowledge*	Curriculum
Support from administrators and coordinating teachers Teachers’ readiness and interest	ii) Teachers’ role	***Nutrition Officer 2*** *The success of the programme comes from the primary support and cooperation of the school. So if the school is very cooperative and supportive in the activities carried out every month, it is constructive....arrange students and places, etc*.	Policy
Active involvement of the PTA* Technical support, materials, and inputs	iii) Strong collaboration with PTA members and agencies	***Teacher 3*** *We do have a combination of PTA committee members who are committed, so we made the garden, and the PTA committee also helped us (arrange) the sports activities for obese children*.	Partnership
The commitment of school canteen operators Ongoing guidance from the teachers and nutrition officers Continuous monitoring	iv) Compliance with the healthy guidelines	***Teacher 3*** *If in terms of the canteen there is no issue. If we are with the canteen operator, we will always discuss ways to improve the menu for kids because our school canteen operator is open and can discuss and tell what could and could not be sold. He tries to fix it as best as he can*.	Policy

**Empirical pattern**			**Theoretical pattern**

**Sub-theme**	**Theme**	**Verbatim**	
	
**Barrier factors**			

Teachers time School rules	i) School system	***Teacher 2*** *It doesn’t matter how much we want to have time because sometimes we have to catch up with other things; we want to catch up with the syllabus. So, for those things, sometimes the teachers themselves don’t have time. We can only do co-curriculum; we can’t interfere with curriculum time*	Policy
Lack of parental support and commitment Constraints in providing healthy meals	ii) Parental awareness	***Nutrition Officer 2*** *Only at home; so when we sometimes tell these students… ok foods like fast food are not suitable for your health, but parents bring them to fast food restaurants, so there’s a clash. It gives the message that healthy eating cannot be implemented in such a correct way. Thus, the implementation of nutrition education in schools is not entirely successful* ***Teacher 3*** *Parents don’t want his/her children to join, and they want their children to focus on learning only. [They think] these activities interfere with their child’s academics*	Partnership
Lack of human resources Time-consuming	iii) Staff availability	***Nutrition Officer 1*** *We lack human resources because there is only one nutrition officer per district. So, if we want to implement and organize this program very well, there must be involvement from the parents and teachers*	Partnership
Lack involvement in activities Unhealthy food habits	iv) Children’s compliance and attitudes	***Teacher 1*** *The attitudes of the students themselves…they don’t want to be interested in joining activities ... because they are already exposed to unhealthy food advertisements that are found in their daily lives*.	Policy (environment)

**Empirical pattern**			**Theoretical pattern**

**Sub-theme**	**Theme**	**Verbatim suggestions**	
	
**Suggestions for improvement**			

Teachers’ role Parents’ role The roles of school canteen operators The roles of nutritionist	i) Strengthening the role of school communities	Teacher 1 *Parents’/teachers’ roles *All activities need to have collaboration with parents so that people can know. We ask to cook healthy food with students and their parents alongside nutrition officers and teachers so that they can bring such knowledge home. All communities need to play a crucial role in obesity management*. Teachers 1 * *Roles of the school canteen operators* *We need to cultivate healthy eating habits, and one that should do so is the canteen operator. They also need to adhere to the selling guideline that we run with the menus that we serve. In addition to selling food for profit, there must also be a culture of sustaining health among students and teachers*.	Partnership
Interaction medium An easy-to-use reference sources Easily accessible reference sources	ii) Integrating nutrition education with technology for the school communities	Nutrition Officer 3 *For parents We need to alert parents about the knowledge and good eating practices for their children. Maybe we need to upload info on social media, or we have to create something that has a link so that parents can be alerted about their children’s nutrition, for example. What can be done to make parents aware that their children are obese or overweight? * *For teachers/school canteen operators* *Teachers need to be guided continuously using new inputs apart from the activities during the programme earlier provided. They may go to the web or any link; maybe the same link as the canteen operator or parents, to get more information to be used as continuous education for children*.	* Emerging new pattern from the curriculum aspect
Integration of intervention components into the curriculum Integration of intervention components into the club activities	iii) Application of intervention elements in the schools’ curricula	***Teacher 1*** *May apply the nutrition intervention component for obese children in other clubs or associations like Puteri Islam. From there, it can involve more students not just focusing on the obese ones and not during obesity interventions only*	Curiculum
Agents of change in the family Agents of change to other students	iv) Students as role models	***Teacher 3*** *Perhaps not all of the 30 children participating are successful in changing their eating habits, but there are one or two who are, and we can use them as examples to other kids and their families*.	Partnership

Note:

* PTA = Parent-Teacher Association
